# Liposomal Resiquimod for Enhanced Immunotherapy of Peritoneal Metastases of Colorectal Cancer

**DOI:** 10.3390/pharmaceutics13101696

**Published:** 2021-10-15

**Authors:** Griffin Pauli, Po-Han Chao, Zhu Qin, Roland Böttger, Suen Ern Lee, Shyh-Dar Li

**Affiliations:** 1 Faculty of Pharmaceutical Sciences, University of British Columbia, Vancouver, BC V6T 1Z3, Canada; griffinpauli96@gmail.com (G.P.); phc@student.ubc.ca (P.-H.C.); qinz0514@gmail.com (Z.Q.); roland.boett@gmail.com (R.B.); leesuenern@hotmail.com (S.E.L.); 2 Institute of Veterinary Immunology & Engineering, Jiangsu Academy of Agricultural Sciences, Nanjing 210014, China

**Keywords:** liposomes, drug delivery, resiquimod, peritoneal metastasis of cancer, immunotherapy, toll-like receptor agonist

## Abstract

Colorectal cancer with peritoneal metastases is currently treated by cytoreductive surgery and locoregional chemotherapeutics. This standard treatment is associated with high morbidity, mortality, and recurrence rate. To augment the existing therapy, we developed a liposome-based delivery system containing 1,2-stearoyl-3-trimethylammonium-propane chloride (DSTAP), a cationic lipid, to localize a toll-like receptor agonist, resiquimod (R848), in the peritoneal cavity (PerC) for enhancing the immune response against cancer that had spread to the PerC. The liposomes delivered by intraperitoneal injection increased peritoneal retention of R848 by 14-fold while retarding its systemic absorption, leading to a 5-fold decreased peak plasma concentration compared to free R848 in mice. Within the PerC, the DSTAP-liposomes were found in ~40% of the dendritic cells by flow cytometry. DSTAP-R848 significantly upregulated interferon α (IFN-α) in the peritoneal fluid by 2-fold compared to free R848, without increasing the systemic level. Combined with oxaliplatin, a cytotoxic agent inducing immunogenic cell death, DSTAP-R848 effectively inhibited the progression of CT26 murine colorectal tumor in the PerC, while the combination with free R848 only showed a mild effect. Moreover, the combination of oxaliplatin and DSTAP-R848 significantly increased infiltration of CD8+ T cells in the PerC compared to oxaliplatin combined with free R848, indicating enhanced immune response against the tumor. The results suggest that DSTAP-R848 exhibits potential in augmenting existing therapies for treating colorectal cancer with peritoneal metastases via immune activation.

## 1. Introduction

The peritoneum is a serous mesothelial lining that encloses the abdominal viscera and acts as a conduit for blood, nervous, and lymphatic structures, in addition to restricting the movement of the internal organs [1]. Metastatic invasion of the peritoneal lining, referred to as peritoneal carcinomatosis (PC), can occur for a variety of abdominal cancers, such as colorectal and ovarian [2]. Peritoneal involvement occurring with colorectal cancer is classified as a late-stage disease and can be difficult to treat. PC of colorectal cancer is currently treated with cytoreductive surgery and hyperthermic intraperitoneal chemotherapy (HIPEC) often composed of folinic acid, 5-fluorouracil, and oxaliplatin. Despite this aggressive approach, the median survival of PC in colorectal cancer patients is only 12 months, and the long-term survival remains negligible, with a high recurrence rate between 20 and 50% [3,4,5,6,7]. The reason behind this poor prognosis is complex; however, the reorganization of the tumor and peritoneal cavity (PerC) microenvironment towards an immunosuppressive phenotype is gaining recognition as a contributing factor [8,9].

During PC tumor progression, there is a reduction in resident T cells and an increase in myeloid-derived suppressor cells (MDSCs) [8]. The combination of decreased cytotoxic lymphocytes and enhanced expression of PD-L1 by MDSCs reduces antitumor immunity by exhausting the infiltrating T cells [10]. This immunosuppressive microenvironment contributes to tumor growth and recurrence [11]. Several approaches have been investigated to reverse immunosuppression in the tumor microenvironment (TME). These include immune checkpoint blockade (e.g., antibodies against PD-1, PD-L1), cancer vaccines, T-cell engagement therapies (e.g., bispecific antibodies, CAR-T cells), and immune response modifiers [12,13]. Among these different approaches, immune response modifiers (IRMs) act to mobilize the immune system and enhance the antitumor immune response [14]. IRMs include cytokines (interferons, IFNs, and interleukins, ILs), Bacillus Calmette–Guérin, and immunomodulatory drugs such as the imidazoquinoline analogs imiquimod, dactolisib, gardiquimod, sumanirole, and resiquimod.

Resiquimod (R848) is a small-molecule agonist for toll-like receptors 7 and 8 (TLR 7/8). R848 exerts its antitumor and antiviral effects through the activation and maturation of dendritic cells (DCs) and inducing secretion of immunostimulatory cytokines such as IFN-α, TNF-α, IL-6, and IL-12 [15,16]. R848 also promotes the maturation of DCs, as evidenced by upregulation of surface markers CD80 and CD86, and mature DCs initiate and sustain T-cell activation [17]. Through the action of cytokine induction and upregulation of cell surface markers, R848 enhances the activation of cytotoxic lymphocytes such as T cells and natural killer (NK) cells while antagonizing immunosuppressive cells such as MDSCs [10]. Overall, R848 can remodel the TME and host response, promoting antitumor immunity [16].

As such, R848 holds significant potential in activating the immune response in the PerC TME to augment the standard therapy for PC of colorectal cancer. However, delivery of R848 needs to be localized in the PerC to effectively activate the TME and reduce systemic and nonspecific immune response such as cytokine storm that causes toxicity [15,16]. We hypothesized that cationic liposomes could increase peritoneal retention of R848 and retard its systemic absorption to improve its local effect and reduce the systemic side effects. When combined with oxaliplatin (OXA), an anticancer drug commonly used for colorectal cancer and an immunogenic cell death inducer through eliciting endoplasmic reticulum (ER) stress [18], the liposomal R848 could activate the TME and enhance the treatment of peritoneal metastases of colorectal cancer.

In this study, we report the development as well as the in vitro and in vivo characterization of liposomal R848. Pharmacokinetics, biodistribution, and immune cell uptake of liposomal R848 were compared with free drug by using ultraperformance liquid chromatography (UPLC) and flow cytometry. Capability of the liposomal R848 in activating the TME and enhancing the therapy against peritoneal metastases of colorectal cancer was compared with free drug in the syngeneic murine colorectal CT26 tumor model. Finally, we compared the TME after different treatments by examining the immune cell composition in the PerC.

## 2. Materials and Methods

### 2.1. Chemical and Reagents

Cholesterol was purchased from Sigma-Aldrich (St. Louis, MO, USA). 1,2-Stearoyl-3-trimethylammonium-propane chloride (DSTAP), 1,2-distearoyl-sn-glycerYo-3-phosphocholine (DSPC), and sodium 1,2-distearoyl-sn-glycero-3-phospho-(1′-rac-glycerol) (DSPG) were purchased from Avanti Polar Lipids (Alabaster, AL, USA). R848 was obtained from Cayman Chemicals (Ann Arbor, MI, USA). Acetic acid, dimethyl sulfoxide, sodium acetate, sodium sulfate, ammonium thiocyanate, polybrene, Dulbecco’s modified Eagle Medium (DMEM), fetal bovine serum (FBS), penicillin, streptomycin, neomycin, and ferric chloride were purchased from Sigma-Aldrich (St. Louis, MO, USA). 1,1′-Dioctadecyl-3,3,3′,3′-tetramethylindocarbocyanine perchlorate (Dil) and 1,1′-dioctadecyl-3,3,3′,3′-tetramethylindotricarbocyanine iodide (DiR) were purchased from Thermo Fisher Scientific (Toronto, ON, Canada). Luciferase plasmid (Plasmid Number: 18964) was obtained from Addgene (Watertown, MA, USA). Turbofectin was ordered from Origene (Rockville, MD, USA). Fluorescence-activated cell-sorting buffer and red blood cell lysis buffer were purchased from Thermo Fisher Scientific (Toronto, ON, Canada). Antibodies and live dead stain (7-AAD) were purchased from Myltenyi Biotec (Auburn, CA, USA).

### 2.2. Liposome Preparation

Lipids dissolved in chloroform/methanol (80:3, *v*/*v*) were mixed according to a fixed molar ratio (X:Y:Z = 2:1:0.8, where X = DSPC, Y = cholesterol, and Z = DSTAP, DSPG, or DSPC), followed by rotary evaporation to prepare a thin lipid film. The lipid film containing ~80 mg of lipids was hydrated with 2 mL of 300 mM ammonium sulfate and then extruded at 60 °C progressively through a series of polycarbonate membranes with pore sizes ranging from 100 to 400 nm using the Mini-Extruder (Avanti, AL, USA) to obtain monodispersed liposomes. Liposomes were dialyzed against 100 mM sodium acetate buffer (pH 5.5) for 24 h, while the buffer was exchanged at 2 h and 4 h. Liposomes were then characterized by size, polydispersity index (PDI), and zeta potential (ZP) using the Zetasizer Nano ZS (Malvern Instruments Ltd. Malvern, UK). The lipid concentration was determined using the Stewart assay, as described below. To prepare DiI or DiR containing liposomes, 1 mol% of DiI/DiR was included in the lipid mixture, followed by the same procedures.

### 2.3. R848 Loading into DSTAP-Liposomes

R848 was added to DSTAP-liposomes at a drug-to-lipid ratio of 1:5 (*w*/*w*), incubated at 60 °C for 30 min, and then cooled on ice for 3 min. The particles were then dialyzed against 5% (*w*/*v*) glucose solution for 24 h with two changes of the dialysate at 2 h and 4 h to remove unencapsulated R848. Lipid and R848 concentrations in the final DSTAP-R848 formulation were measured using the Stewart assay and ultra-high-performance liquid chromatography (UPLC), respectively, as described below. Particle size, PDI, and ZP of DSTAP-R848 were measured by the Zetasizer Nano ZS.

### 2.4. Stewart Assay

A 5 µL volume of liposomes was thoroughly mixed with 1995 µL of chloroform and 2 mL of ferrothiocyanate solution (0.1 M ferric chloride hexahydrate, 0.4 M ammonium thiocyanate), followed by centrifugation at 300× *g* for 10 min (Sorvall Legend Micro 21 R, Thermo Fisher, Toronto, ON, Canada). The optical density of the supernatant at 485 nm was determined using a spectrophotometer (SynergyMx, BioTek, Winooski, VT, USA). The result was compared to a calibration curve to estimate the phospholipid concentration.

### 2.5. UPLC

Liposomes were dissolved in methanol (1:10, *v*/*v*) and sonicated for 10 min, and 10 µL of the sample was injected into a Waters ACQUITY UPLC H-Class system (Milford, MA, USA). The samples were separated on a Waters Acquity BEH-C18 column (particle size: 1.7 µm, inner diameter: 2.1 mm, length: 50 mm) at a flow rate of 0.4 mL/min. The mobile phase consisted of solvent A (0.1% formic acid in water, *v*/*v*) and solvent B (aqueous 90% acetonitrile containing 0.1% formic acid, *v*/*v*). The following gradient was applied: 3.0 min: A/B (95/5), 11.0 min: A/B (47/53), 12 min: A/B (5/95), 13.0 min: A/B (5/95), 13.5 min: A/B (95/5), 15.0 min: A/B (95/5). The samples were detected via a QDa mass spectrometer (Waters) operated in the positive ionization mode. Ionization was carried out at a source temperature of 450 °C using a cone voltage of 25 kV. The R848 concentration was determined by integrating the single-ion recognition (SIR) peak for the singly charged molecular ion as *m*/*z* 315 using mefloquine as internal standard with the SIR peak at *m*/*z* 379. Data were analyzed using the Empower 3.0 software (Waters).

### 2.6. Cell Culture

RAW 264.7 murine macrophage and CT-26 murine colorectal cells were obtained from American Type Cell Collection (Gaithersburg, ML, USA) and propagated in DMEM supplemented with 5% FBS and 1% penicillin/streptomycin. Generation of the luciferase-expressing CT-26 cells (CT-26-Luc) was achieved by using a lentiviral vector containing a luciferase gene with neomycin resistance. Luciferase-expressing cells were selected under a culture medium containing 1 mM of neomycin.

### 2.7. Mice

Female BALB/C mice (age: 6–8 weeks) were purchased from Charles River Laboratories (Wilmington, MA, USA) and housed according to the institutional guidelines of the University of British Columbia (UBC). Food and water were provided ad libitum. All animal experiments were conducted with the approved Protocol Number A18-0177 at UBC.

### 2.8. Pharmacokinetic and Biodistribution

Free R848 or DSTAP-R848 was injected intraperitoneally (IP) into mice at a dose of 1 mg of R848/kg. At different time points (15 min, 1 h, 4 h, and 24 h), blood was collected via cardiac puncture, followed by euthanization and the peritoneum fluid (PF, original volume: ~100 µL) was expanded and collected after IP instillation with 2 mL of phosphate-buffered saline (PBS). Plasma was obtained by centrifuging blood (5000 rpm, 10 min) immediately after collection in an ethylenediaminetetraacetic acid (EDTA) collection tube (Microvette, Sarstedt, Nümbrecht, Germany), and IP cells (i.e., cells in PF) were isolated from PF by centrifugation (1500 rpm, 5 min).

R848 in plasma, PF, and IP cells was measured by UPLC. Briefly, the IP cell pellet was suspended in 50 μL of 1% (*v*/*v*) aqueous Triton-X 100 solution, incubated for 15 min on ice, followed by centrifugation (12,500 rpm, 10 min) to harvest the supernatant. Plasma, PF, or IP cell lysate (all 45 μL) was mixed with 30 μL of mefloquine solution (1 mg/L in 90% (*v*/*v*) aqueous acetonitrile) and 270 μL of cold 90% (*v*/*v*) aqueous acetonitrile. The mixture was vortexed for 30 s, placed on ice for 30 min, and centrifuged (12,500 rpm, 5 min). The supernatant (300 μL) was collected, lyophilized, and rehydrated in 60 μL of 4.5% (*v*/*v*) aqueous acetonitrile containing 0.1% formic acid. A 10 µL volume of the sample was then analyzed by the UPLC method, as described above.

### 2.9. Cytokine Quantification

IFN-α in the plasma and PF were measured by enzyme-linked immunosorbent assay (ELISA) using the eBioscience kit (Thermo Fisher, Toronto, ON, Canada) following the manufacturer’s instructions.

### 2.10. Cellular Uptake by IP Cells

Mice (*n* = 3) were IP inoculated with 2 × 10^5^ CT-26-Luc cells on Day 4. On Day 0, mice were IP injected with 15 mg/kg luciferin and then imaged using the Lumina IVIS system (PerkinElmer, Waltham, MA, USA) to confirm the presence of tumors in the peritoneal cavity. Mice then received 6 mg/kg OXA via IP injection. The following morning, each mouse received an IP dose of DiI-labeled DSTAP-liposomes at 10 mg lipid/kg. After 1 h, mice were sacrificed, and IP was instilled with 5 mL of ice-cold PBS for PF collection. The PF was centrifuged to obtain IP cells, which were resuspended in a staining buffer (PBS containing 1% bovine serum albumin and 0.05% sodium azide), counted, dispensed into a microtube, and stained with various antibodies (anti-CD45, anti-CD11b, anti-CD11c, anti-F4/80, anti-CD335, anti-CD3, anti-CD8, and anti-EPCAM, BD Life Sciences, Franklin Lakes, NJ, USA) for 30 min on ice in the dark. The cells were centrifuged, resuspended in the fluorescence-activated cell-sorting buffer containing 1 mg/mL propidium iodide, and analyzed by Cytoflex LX (Beckman Coulter, Brea, CA, USA).

### 2.11. In Vivo Efficacy

Mice were IP inoculated with 2 × 10^5^ CT-26-Luc cells on Day 4. On Day 0, mice were IP injected with 15 mg/kg luciferin and then imaged using the Xenogen IVIS system to confirm the presence of tumors in the peritoneal cavity. Mice with roughly equal levels of bioluminescent tumor expression were then assigned randomly to three groups. On Day 0, all mice received an IP dose of 6 mg/kg oxaliplatin (OXA). On Day 1, mice received either PBS, free R848, or DSTAP-R848 at 8 mg R848/kg. Mice were then imaged regularly to monitor tumor progression and euthanized on Day 13 as the study endpoint. Overall health and body weight of the mice were recorded daily to monitor the toxicity of the treatments.

### 2.12. Immune Cell Uptake and Composition

Following the same protocol as described in the efficacy study, mice were sacrificed on Day 10, and their IP cells were collected. Similar to the uptake study, the IP cells were immediately stained with antibodies, including anti-mouse CD3e-VioBright FITC, CD4-PE, CD8a-VioBlue, CD45-APC-Vio770, CD11b-VioGreen, CD11c-PE-Vio770, CD80-VioBright 515, CD86-PE, Anti-Ly-6C-APC, and Anti-CD335-PerCPCy5. After staining with live dead marker 7-AAD-Y675-PC, cells were submitted to fluorescence-activated cell sorting (FACS) to determine the levels of immune cells infiltrating the TME. The FACS data were analyzed using FlowJo™ (Ashland, OR, USA).

### 2.13. Statistical Analysis

All data are expressed as mean ± SD or mean ± SEM. Statistical analysis was conducted with the two-tailed unpaired *t*-test for two-group comparison or one-way ANOVA, followed by Tukey’s multiple comparison test using GraphPad Prism 7 (San Diego, CA, USA) for three or more groups. A difference of *p* < 0.05 was considered statistically significant.

## 3. Results

### 3.1. Selection and Characterization of Liposomal Formulations

To test our hypothesis that cationic liposomes would exhibit enhanced IP retention, we prepared three liposomal formulations displaying different surface charge characteristics corresponding to the incorporation of a neutral (DSPC), cationic (DSTAP), or anionic (DSPG) lipid and compared their retention in the PerC after IP delivery (Table S1). To track their IP retention, we spiked 1 mol% of DiR, a fluorescent dye, in the formulations to allow in vivo tracking of these liposomes by fluorescence imaging. As shown in Supplementary Table S1, the three formulations displayed comparable size (~80 nm) and PDI (<0.1) but distinct zeta potential (ZP). The DSTAP-, DSPC-, and DSPG-liposomes labeled with DiI or DiR exhibited ZPs of 51.3, 7.2, and −42.9 mV, respectively. Following IP delivery, the positively charged DSTAP-liposomes showed the most significant IP retention with minimal fluorescence detected outside the peritoneal region even after 24 h (Supplementary Figure S1). In contrast, 4 h after IP injection, both neutral DSPC- and anionic DSPG-liposomes were detected outside the abdominal region. The cationic DSTAP-liposomes might rapidly interact with the negatively charged cell membrane in the PerC and remain in that compartment. We also incubated these three formulations with RAW 264.7 cells, a model line for immune cells, to compare their intracellular delivery. As shown in Supplementary Figure S2, DSTAP-liposomes were more effectively internalized compared to the other liposomal formulations and predominantly localized in the lysosomes (Lysotracker Green +), where the TLR7/8 is expressed. The intracellular fluorescence of DSPC- and DSPG-liposomes was significantly lower and largely not colocalized with the lysosomes. These preliminary data support the use of cationic DSTAP-liposomes for delivering R848, a TLR7/8 agonist, to the IP immune cells for enhanced immunotherapy of PC. We thus selected DSTAP-liposomes as the delivery vehicle for R848.

To achieve a high loading efficiency, R848 was actively loaded into the liposomes using the solvent-assisted active loading technology (SALT) [19], rendering an encapsulation efficiency of 80.2 ± 3.2%. The final DSTAP formulation was dialyzed against 5% glucose to remove any unincorporated R848. The DSTAP-liposomal formulation for R848 (DSTAP-R848) was characterized for the drug content, lipid concentration, size, polydispersity index (PDI), and ZP, and the results are shown in Table 1. DSTAP-R848 liposomes displayed a mean diameter of ~100 nm and a relatively monodispersed population as the PDI was below 0.1, with a positive ZP of 43.0 mV.

### 3.2. Pharmacokinetic Profile of DSTAP-R848

**Figure 1:**
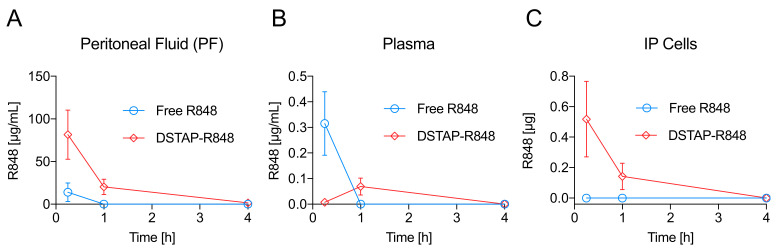
R848 concentrations in the (**A**) peritoneal fluid, (**B**) plasma, and (**C**) IP cells after IP administration (1 mg/kg) of free R848 or DSTAP-R848 in mice. (Data = mean ± SD, *n* = 5).

We then examined the pharmacokinetic and biodistribution profiles of free R848 and DSTAP-R848 in a mouse model to compare their IP retention. After IP administration of either free drug or DSTAP-R848, we collected blood and peritoneal fluid (PF) at various time points. We also retrieved IP cells from the PF to study the immune cellular uptake of the two formulations. As shown in Figure 1A and Table 2, mice treated with DSTAP-R848 displayed ~14-fold increased dose exposure in the PF (calculated as the area under the curve, AUC_0.25−4h_) relative to free drug, which was removed from the peritoneum within 1 h. Accordingly, within 15 min after injection, free R848 was absorbed into the plasma, achieving a C_max_ of 0.3 µg/mL, while the plasma C_max_ in the DSTAP-R848 group occurred at 1 h with a 5-fold reduced concentration (Figure 1B). However, plasma AUC_0.25−4h_ was comparable between the two groups (Table 2). This is likely due to a delayed absorption effect occurring with DSTAP-R848. The drug exposure ratios (PF/plasma) for DSTAP-R848 and free R848 were ~680 and ~45, respectively (Table 2), indicating preferential IP retention of DSTAP-R848 compared to free R848. Lastly, R848 in the IP cells collected from the PF was under the detection limit in the free drug group, while a significant amount of R848 (0.1–0.5 µg) was measured in the DSTAP-R848 group during the first hour after IP injection (Figure 1C). Overall, the data indicate that free R848 was rapidly absorbed from the peritoneum into the blood circulation, with poor IP retention and little uptake by the IP cells. In contrast, DSTAP-R848 effectively remained in the peritoneum and displayed increased delivery to the IP cells compared to free R848, with delayed systemic absorption, which could lead to reduced global TLR7/8 activation.

Although the peritoneum is a relatively confined microenvironment, there is a continuous exchange between blood and PF. It was likely that the IP delivered free R848 did not stay in the peritoneum, but was rapidly absorbed into the blood, causing systemic toxicity. As calculated with data from Figure 1, it is confirmed that a significant portion (25%) of the IP-injected free R848 was absorbed into the plasma within 15 min, and 1 h after injection, the drug concentration in the PF was under the detection limit (<0.5 ng/mL). Encouragingly, DSTAP-R848 displayed increased IP retention, effective uptake by the IP immune cells, and decreased peak plasma concentrations of R848, suggesting that this liposomal formulation was able to localize R848 in the PerC to enhance the local immune activation.

### 3.3. Delivery into Peritoneal Immune Cells

As we demonstrated that the DSTAP formulation was able to increase the delivery of R848 to the IP cells relative to free drug, we sought to further characterize which immune cells in the peritoneum were responsible for this uptake. DSTAP-liposomes were labeled with 1 mol% of DiI and IP injected into CT-26-Luc tumor-bearing mice (*n* = 3) to mirror the efficacy study. The PF was retrieved 1 h after injection, and the IP cells were collected, stained using fluorescent antibodies, and analyzed by flow cytometry.

**Figure 2:**
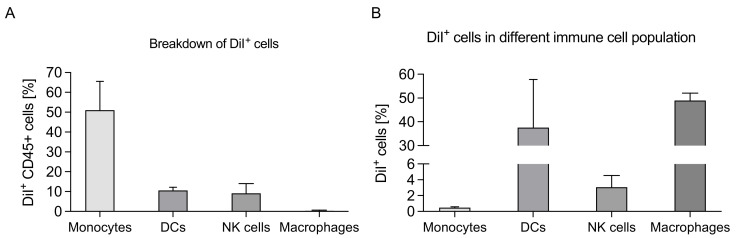
Delivery of DSTAP-liposomes to immune cells in the peritoneal cavity. (**A**) Percentage of total DiI+ cells by cell type (data = mean ± SD, *n* = 3). (**B**) DiI+ cell population in each cell type in the peritoneal cavity (data = mean ± SD, *n* = 3).

After selecting singlets using forward scatter by side scatter, we focused on live and DiI+ cells and then examined the immune cell populations (Figure 2 and Figure S3). We found that the monocyte population (CD45^+^, CD11b^+^, CD3^−^, CD19^−^, CD11c^−^, F4/80^−^) accounted for ~50% of the total DiI+ IP cells (Figure 2A). In addition, approximately 10% and 8% of the DiI+ IP cells were myelogenous DCs (CD45^+^, CD11b^+^, CD11c^+^) and NK cells (CD45^+^, CD11b^+^, CD11c^−^, NKp46/CD335^+^), respectively (Figure 2A) [20]. We further investigated the DiI+ populations using a macrophage surface marker F4/80 and found that only 0.37% of the DiI+ cells were macrophages. Uptake by EpCAM+ tumor cells and other immune cells such as T cells and B cells was not detectable (data not shown).

To further characterize liposomal uptake, we compared the DiI+ population as a percent of the total population of each cell type in the PerC (i.e., both DiI+ and DiI− live cells). DCs and macrophages showed the highest percent uptake at approximately 40% and 50% of the cell type in the PerC being DiI+, respectively (Figure 2B). It has been shown that cationic nanoparticles can more effectively interact with DCs and macrophages compared to other immune cells [21,22] because both cell types exhibit increased phagocytic activity [23,24]. Although ~50% of the DiI+ IP cells were monocytes, only <1% of the total IP monocytes were associated with DSTAP-liposomes, suggesting that the monocyte uptake was non-specific and was due to the large population in the PF of tumor-bearing mice [25]. Overall, the uptake data indicate that DSTAP-liposomes were selective towards DCs compared to other cell types. DC is the mediator of T cell immunity, and focusing delivery of R848 to DCs could promote this pathway for antitumor effects, although R848 was also shown to directly activate other immune cells such as NK cells and macrophages to induce antitumor activity [26,27,28].

### 3.4. Cytokine Stimulation in the Peritoneum

The immune cell uptake data showed that the DSTAP-liposomes selectively deliver to DCs in the PerC. Stimulation of the TLR7/8 in DCs by R848 has been shown to induce the production of IFN-α, which can prime, expand, and sustain the cytotoxic CD8 T cell [17,29,30]. In addition, IFN-α is also related to surface upregulation of CD83, 80, 86, and 40 on DCs, leading to maturation, which contributed to enhanced antigen presentation [17,31,32]. Therefore, IFN-α has been demonstrated to turn “cold” (immunosuppressed) tumors into “hot” (immunoactive) tumors, leading to improved disease prognosis [33,34].

**Figure 3:**
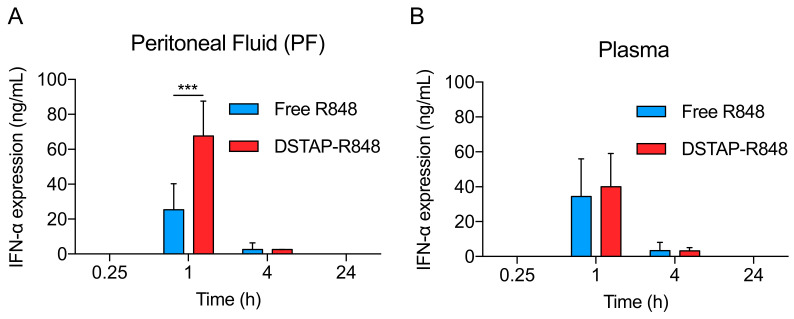
IFN-α levels in the (**A**) peritoneal fluid, (**B**) plasma after IP administration of free R848 and DSTAP-R848 in mice. (Data = mean ± SD, *n* = 5, ***: *p* < 0.001).

We then selected IFN-α and further investigated its protein levels in the PF (local) and plasma (systemic) by ELISA to compare the immunological effect of PBS, free R848, and DSTAP-R848 after IP delivery. As shown in Figure 3A, IFN-α could only be detected between 1 h and 4 h, and DSTAP-R848 treatment significantly increased IFN-α level in the PF by 3-fold compared to free drug at 1 h. The data are consistent with the IP retention (Figure 1) results, supporting that DSTAP-R848 improved the IP retention and local immunological response in the PerC. In terms of the plasma levels, the IFN-α secretion was comparable in free drug and DSTAP-R848 groups (Figure 3B). Overall, the data indicate that the local effect (in the PF) induced by DSTAP-R848 was increased compared to free R848 with a similar systemic effect (in the plasma). This finding is supported by the pharmacokinetics and biodistribution data in Figure 1, showing that IP delivered DSTAP-R848 produced a comparable AUC in the plasma but 14-fold increased AUC in the PF relative to free drug.

### 3.5. Antitumor Efficacy

**Figure 4:**
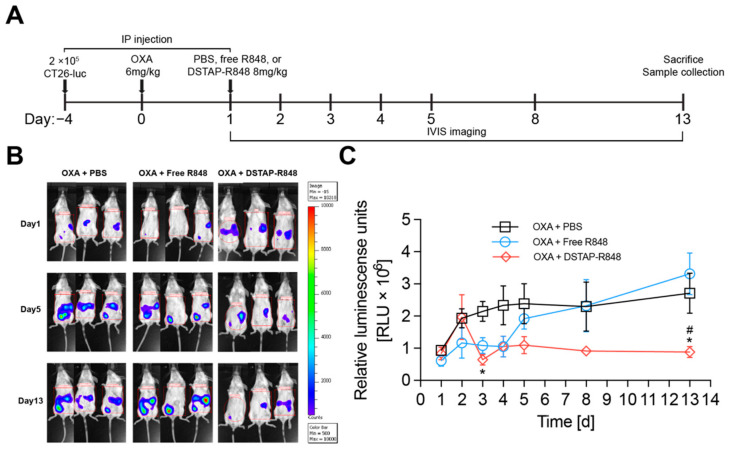
In vivo efficacy against a PC model of CT-26-Luc in female BALB/C mice. (**A**) Treatment schedule diagram. (**B**) Representative bioluminescent images of mice in different treatment arms. (**C**) Quantitative data of CT26-Luc bioluminescence in mice after different treatments. (Data = mean ± SEM, *n* = 5−6) *: *p* < 0.05 relative to PBS and #: *p* < 0.05 relative to free R848.

The goal of this study was to develop an improved delivery system enabling a new treatment to augment existing therapies for peritoneal metastases of colorectal cancer. We then compared the antitumor effect of different R848 formulations (free R848 and DSTAP-R848) in combination with oxaliplatin (OXA), a cytotoxic agent that is commonly used for treating colorectal cancer and can induce immunogenic cell death [35]. To ensure this combinational effect was synergistic rather than additive, we also demonstrated that there was no obvious cytotoxicity observed in vitro when CT26 cells were treated with either free R848 or empty DSTAP liposomes (Figure S4). As shown in Figure 4A, mice were first inoculated with 2 × 10^5^ CT-26-Luc cells/mouse via IP injection on Day 4, and then administered IP OXA at 6 mg/kg on Day 0, followed by an IP injection of PBS, free R848, or DSTAP-R848 on Day 1 at 8 mg R848/kg. Tumor progression in the peritoneum was monitored and quantified by bioluminescence imaging. As shown in Figure 4B, mice treated with OXA alone displayed rapid tumor progression, while those treated with OXA + free R848 showed an initial reduction in tumor growth on Days 3–4, but the tumors quickly regrew and spread on Days 5–13. On Day 13, there was no difference between the OXA alone and OXA + free R848 groups (Figure 4C). The initial inhibition of tumor progression in the OXA + free R848 group could be due to that free R848 induced IFN-α that exhibited a significant antitumor effect. However, the IFN-α level decreased rapidly (Figure 3), and the tumors returned. In contrast, OXA + DSTAP-R848 started showing antitumor efficacy on Day 3, and the PC was consistently inhibited on Days 3–13, and on Day 13, the tumor load measured by bioluminescence was 3-fold significantly lower than the other two groups. Furthermore, data in Supplementary Figure S3 indicate that neither R848 nor DSTAP-liposomes had a direct effect against CT-26 cancer cells. Collectively, the data suggest that OXA + DSTAP-R848 induced prolonged antitumor activity, possibly due to an enhanced immune response. We also compared the impact of the treatments on the health of mice by monitoring their activity, appearance, posture, breathing, hydration, and body weight. There was no significant change in health in each group within one week after the treatments, suggesting that none of these therapies caused significant toxicity (data not shown).

### 3.6. Modulation of Immune Cell Composition in PerC

**Figure 5:**
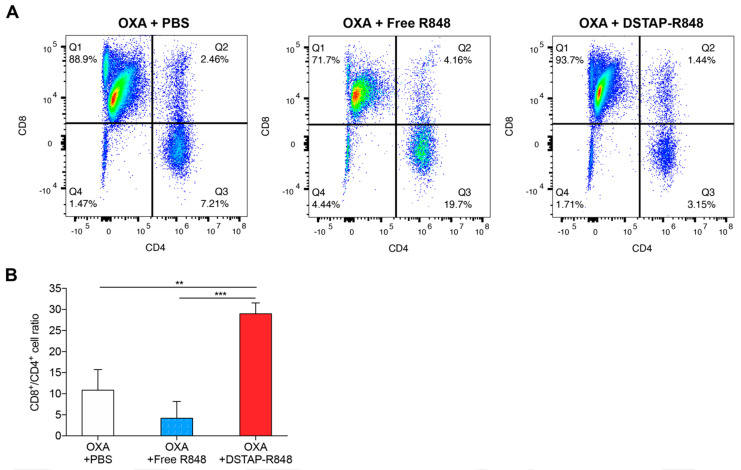
Change in CD4+ and CD8+ T-cell population in the peritoneal fluid after different treatments. (**A**) Representative flow cytometry plots showing the T-cell profiles. (**B**) Summary of CD8/CD4 ratio in each treatment group (data = mean ± SD, *n* = 3). **: *p* < 0.01, ***: *p* < 0.001, n.s.: no significance.

The cytokine data (Figure 3) and efficacy results (Figure 4) suggest that OXA + DSTAP-R848 induced antitumor immunity. We then examined the change in immune cell populations in the PerC microenvironment (i.e., PF) after different treatments (OXA + PBS, OXA + free R848, OXA + DSTAP-R848). Following the same protocol described in the efficacy study, mice were sacrificed on Day 10, and the cells collected from the PF were stained using fluorescent antibodies and analyzed by FACS to quantify key immune cells, including CD4 T cell, CD8 T cells, NK cells, monocytes/macrophages, MDSCs, and DCs. Among these different immune cells, only T cells (CD3+) displayed significant changes among different treatment groups (Figure 5 and Figure S5). Figure 5A shows the T-cell profiles in the PF from one representative mouse in each treatment arm: in the OXA + PBS group, 87.5% and 7.92% of the T cells (CD3+) were CD8+ and CD4+, respectively. In the OXA + free R848 group, the CD8+ population decreased to 71.0%, and the CD4+ population increased to 19.8%. In the OXA + DSTAP-R848 group, the CD8+ T cells increased to 93.5%, while the CD4+ T cells decreased to 3.1%. We then analyzed the CD8:CD4 ratios for each arm (*n* = 3), and as shown in Figure 5B, the CD8:CD4 ratios in the OXA + PBS, OXA + free R848, and OXA + DSTAP-R848 groups were 10.9, 4.2, and 29.0, respectively. While there were no significant differences between the PBS and free R848 groups, the DSTAP-R848 group displayed a 2.5- and 5-fold increased CD8/CD4 ratio relative to the PBS and free R848 groups, respectively. DSTAP-R848 was shown to boost CD8+ T-cell infiltration to the TME, which may be explained by the increased level of IFN-α as shown in Figure 3. It has been reported that IFN-α can cross-prime CD8+ T cells to promote tumor killing [36,37,38]. CD8+ T cells have been known to be the major effector cells that eliminate cancer cells [39]. On the other hand, CD4+ T cells can be converted to immunosuppressive phenotypes through tumor hypoxia and tumor-derived cytokines [40,41,42]. Hence, the increased ratio of CD8+/CD4+ cells in the OXA + DSTAP-R848 group was consistent with the enhanced antitumor efficacy.

**Figure 6:**
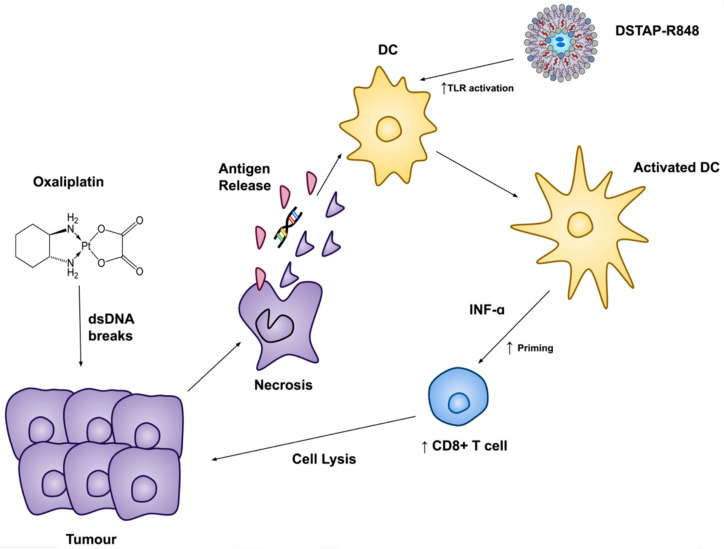
Proposed mechanism of the combination of oxaliplatin and DSTAP-R848.

With the supporting data described above, we have proposed a plausible mechanism of how DSTAP-R848 exerted its antitumor effect in combination with OXA (Figure 6). IP-injected OXA induced immunogenic cell death of the CT26-Luc cells in the PerC, releasing tumor antigens in the PF. DSTAP-R848 delivered by IP displayed increased IP retention and was effectively internalized by DCs for the TLR7/8 activation. Both tumor antigen release and TLR activation led to the maturation of DCs, resulting in increased production of IFN-α and priming of T cells toward CD8+ cytotoxic T cells for tumor killing. Future studies will focus on testing this hypothesis.

This study was focused on developing an improved formulation for R848 to augment the existing therapy (i.e., OXA) for peritoneal metastases of colorectal cancer and has yielded promising results. Follow-up studies will focus on administering multiple doses of OXA + DSTAP-R848 to induce profound tumor regression and examining the development of tumor-specific immunity.

## 4. Conclusions

We developed and characterized a DSTAP-R848 formulation for augmenting PC therapy in combination with OXA. The DSTAP-liposomes largely focused R848 to the DCs in the PF while delaying its systemic absorption. As a result, DSTAP-R848 increased the release of IFN-α in the TME. DSTAP-R848 promoted T-cell priming to CD8+ cytotoxic T cells and potentiated the antitumor efficacy against a syngeneic PC model in combination with OXA. This new combination therapy was shown safe and effective in a mouse model, providing a robust immunochemotherapeutic option for peritoneal metastasis of colorectal cancer.

## Figures and Tables

**Table 1:** Characterization of DSTAP-R848. Data = mean ± SD, *n* = 3.

R848 Conc. (mg/mL)	Total Lipid Conc. (mg/mL)	Size (nm)	Polydispersity Index (PDI)	Zeta Potential (mV)
1.76 ± 0.64	9.59 ± 2.06	104.6 ± 7.2	0.084 ± 0.001	43.0 ± 5.3

**Table 2:** AUCs of R848 concentrations displayed in Figure 1.

Parameter	Free R848	DSTAP-R848	Significance
AUC_0.25−4h_ (h • µg/mL) in peritoneal fluid	5.29 ± 4.10	71.06 ± 17.70	*p* < 0.005
AUC_0.25−4h_ (h • µg/mL) in plasma	0.12 ± 0.05	0.13 ± 0.05	*p* > 0.05
AUC_0.25−4h_ (h • µg) in IP cells	0	0.46 ± 0.16	*p* < 0.005

## Data Availability

The data presented in this study are available on request from the corresponding author.

## Supplementary Materials

The following are available online at https://www.mdpi.com/article/10.3390/pharmaceutics13101696/s1, Table S1. Characterization of fluorescently labeled liposomes, Figure S1: The distribution of DiR-labeled liposomes incorporating DSPC, DSPG or DSTAP the mouse peritoneal cavity over 24 h (*n* = 3), Figure S2: Microscopic images of Raw 264.7 cells after incubation with DiI-liposomes for 24 h, Figure S3: Representative of uptake of DiI liposomes in immune cells in peritoneal cavity. Figure S4: Cell viability of CT26 after 24 h treatment of free R848 or empty DSTAP liposome, Figure S5: Relative quantification NK cells, MDSCs, CD11b+ macrophages, and CD11c+ DCs in CD45+ leucocytes recovered from the peritoneal fluid in each treatment group.
