# Supplementary material for: Liposomal Resiquimod for Enhanced Immunotherapy of Peritoneal Metastases of Colorectal Cancer
Source: Pharmaceutics. 2021 Oct 15;13(10):1696. doi: 10.3390/pharmaceutics13101696 (PMC8540734; doi:10.3390/pharmaceutics13101696)
Supplement: Supplementary file 1 [file pharmaceutics-13-01696-s001.zip › pharmaceutics-1338845-supplementary.pdf]

pharmaceutics

MDPI

Supplementary Materials: Liposomal Resiquimod for Enhanced Immunotherapy of Peritoneal Metastases of Colorectal Cancer

Griffin Pauli, Po-Han Chao, Zhu Qin, Roland Böttger, Suen Ern Lee and Shyh-Dar Li

Table S1. Characterization of fluorescently labeled liposomes.

Liposomal formulation	Size (nm)	PDI	Zeta Potential (mV)
DSPC/ DSTAP/ Chol/DiI (52/21/26/1, mol)	79.57 ± 6.12	0.047 ± 0.017	51.3 ± 2.9
DSPC/Chol/DiI (73/26/1, mol)	80.79 ± 19.31	0.063 ± 0.030	7.2 ± 2.0
DSPC/ DSPG/ Chol/DiI (52/21/26/1, mol)	84.57 ± 5.70	0.016 ± 0.009	−42.9 ± 0.4
DSPC/Chol/DSTAP/DiR (52/21/26/1, mol)	79.57 ± 0.01	0.047 ± 0.006	51.30 ± 1.71
DSPC/Chol/DiR (73/26/1, mol)	80.79 ± 0.59	0.063 ± 0.035	7.19 ± 0.47
DSPC/Chol/DSPG/DiR (52/21/26/1, mol)	77.92 ± 0.84	0.016 ± 0.014	−42.90 ± 1.32

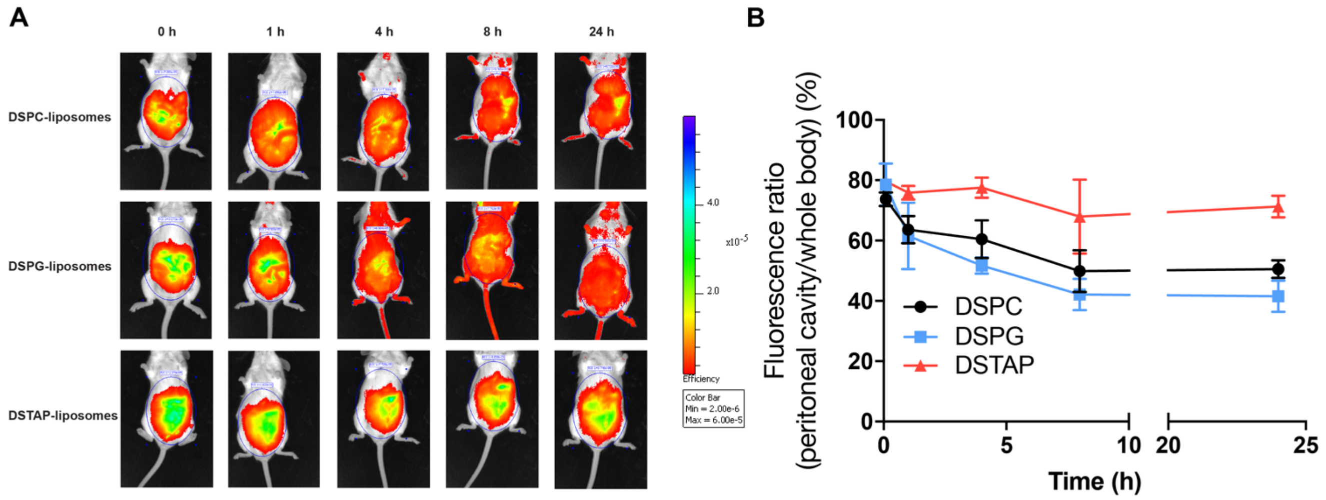

Figure S1. The distribution of DiR-labelled liposomes incorporating DSPC, DSPG or DSTAP the mouse peritoneal cavity over 24 h (*n* = 3). (**A**) Representative images (**B**) Fluorescence ratios of the images over time (Data=mean ± SD, *n* = 3). Fluorescence ratio = fluorescence in the peritoneal cavity divided by whole body fluorescence × 100%.

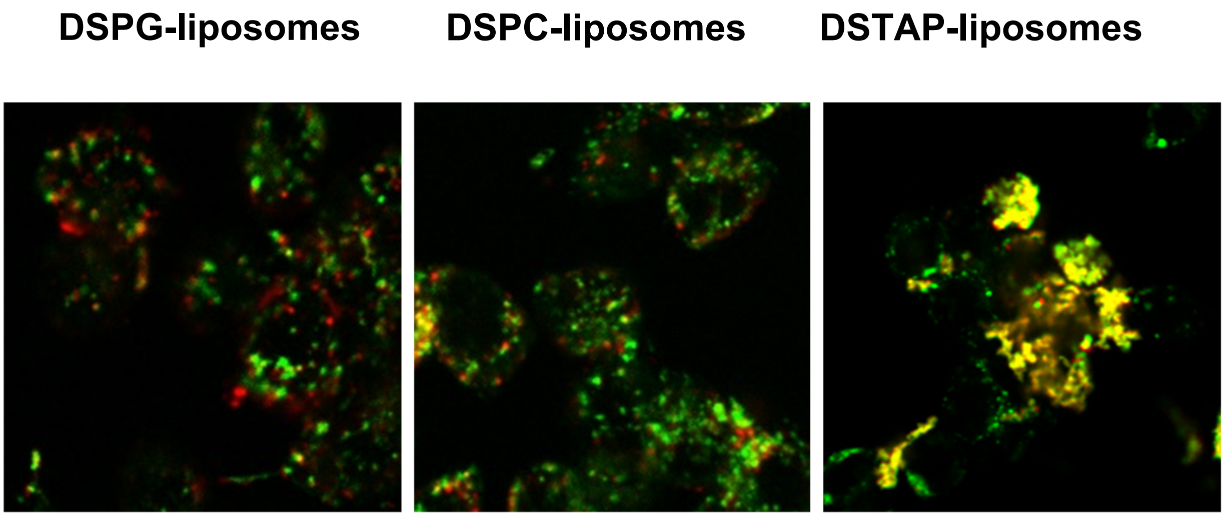

Figure S2. Microscopic images of Raw 264.7 cells after incubation with DiI-liposomes for 24 h. Cells were also treated with Lysotracker Green to reveal association between liposomes (red) and lysosomes (green). Overlay of liposomes (red) and lysosomes (green) is shown in yellow.

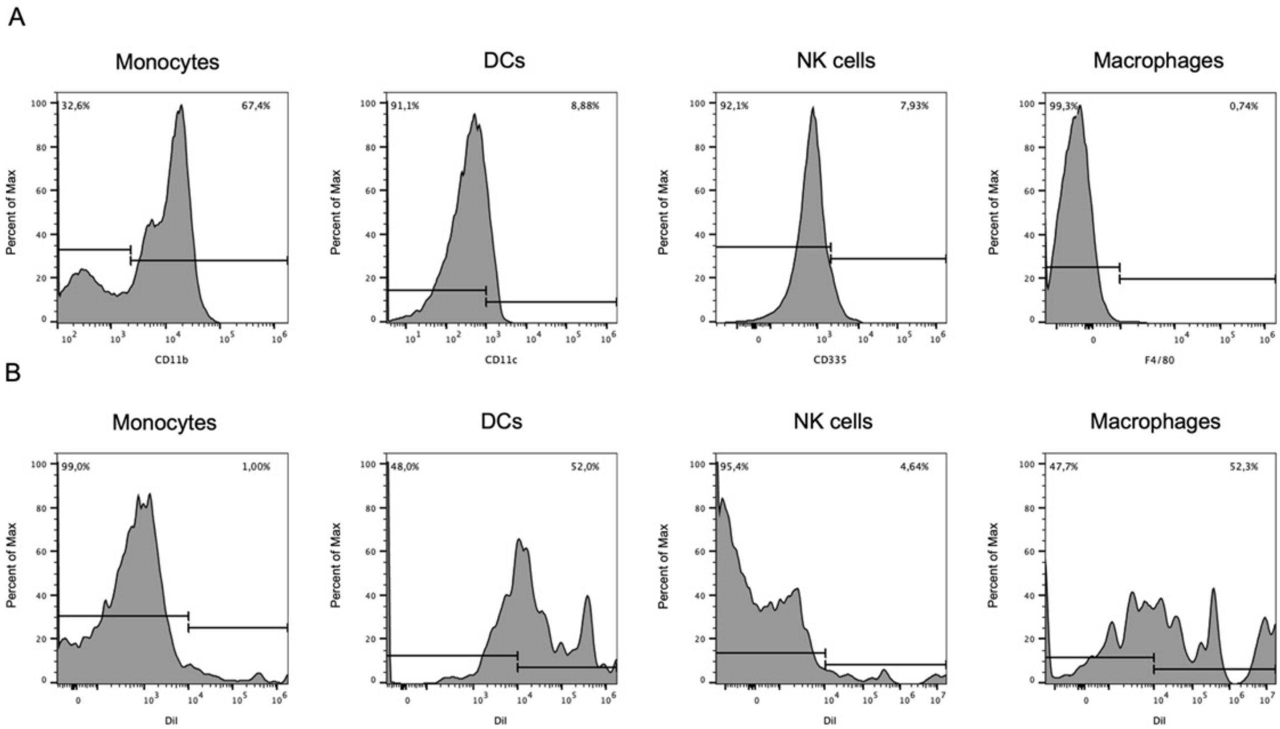

Figure S3. Representative of uptake of DiI liposomes in immune cells in peritoneal cavity (**A**) Percentage of total DiI+ cells by cell type. (**B**) DiI+ cell population in each cell type in the peritoneal cavity.

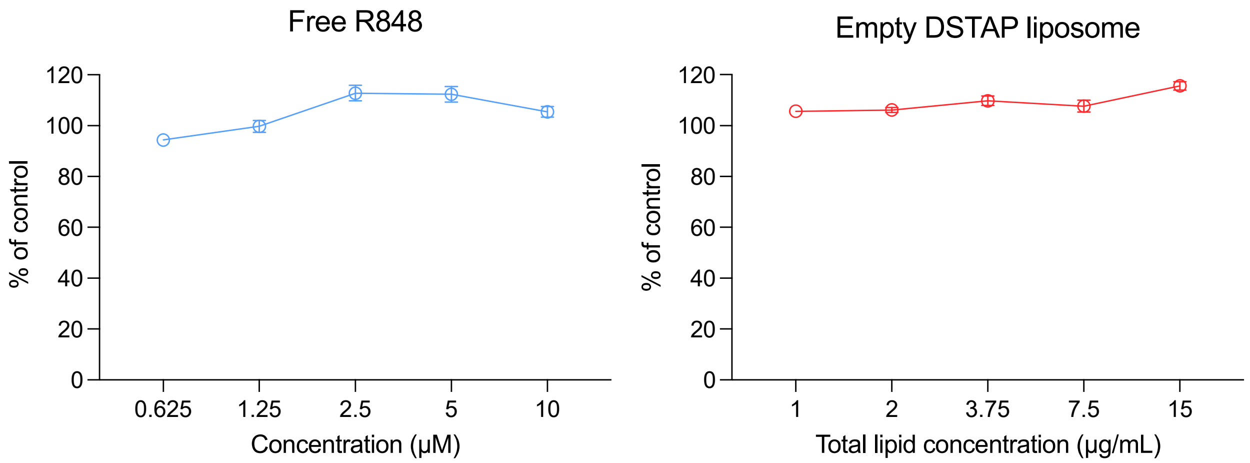

Figure S4. Cell viability of CT26 after 24h treatment of free R848 or empty DSTAP liposome. The concentrations of the liposome used were determined by 1:5 drug-to-lipid ratio. Data=mean ± SEM, n = 12.

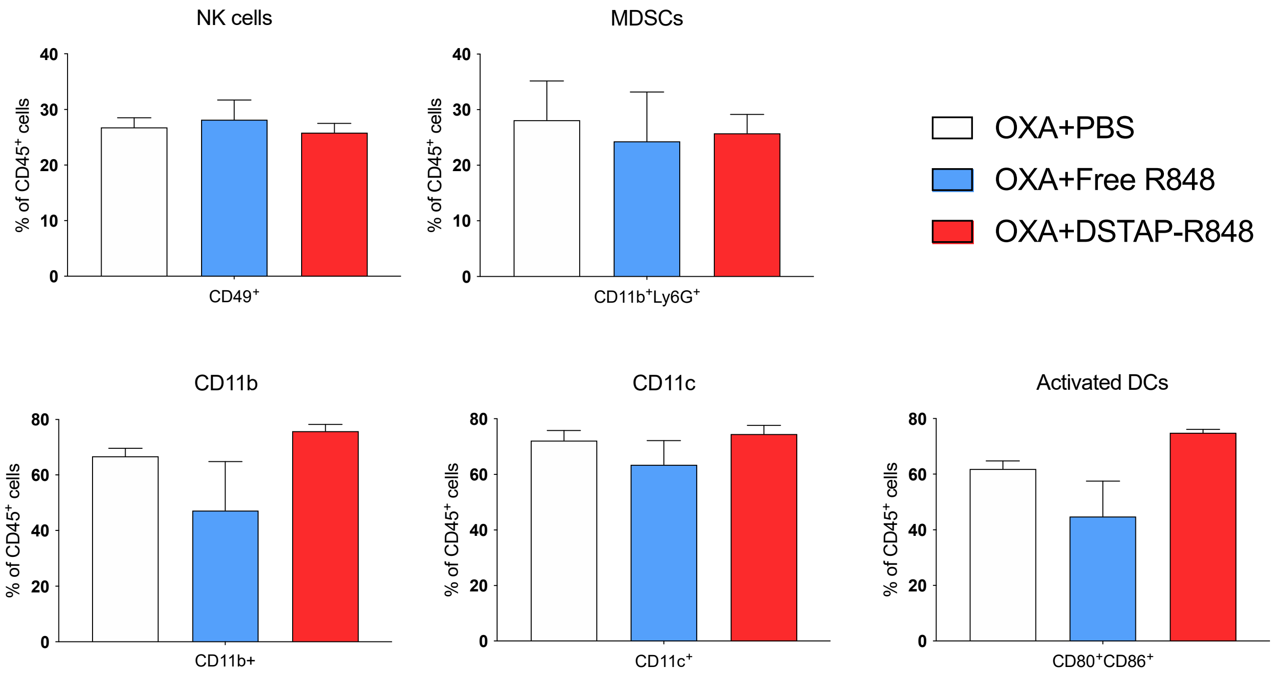

Figure S5. Relative quantification NK cells, MDSCs, CD11b+ macrophages, and CD11c+ DCs in CD45+ leucocytes recovered from the peritoneal fluid in each treatment group. Data=mean ± SD, n = 3. There was no significant difference among these three treatm.
